# Elements of Dental Materia Medica and Therapeutices

**Published:** 1883-01

**Authors:** 


*Elements of Dental Materia Medica and Therapeutics with Pharmacopœa:*—By James Stocken, L. D, S. England. Assisted by Thomas Gaddess, L. D. S. England and Edin. Publishers: P. Blackiston, Son & Co. Philadelphia. 1882.

The recent advancement of Dental Pharmacology, and the demand for such a work, has caused the Author to present a third Edition, in which the old matter has been revised, several new preparations inserted. as well as other important additions made, all of which enhance the value of this work very materially. The Index of Diseases is also an interesting and important addition, and the present volume is one-fifth larger than the former edition.

